# Formulation and In Vivo Pain Assessment of a Novel Niosomal Lidocaine and Prilocaine in an Emulsion Gel (Emulgel) of Semisolid Palm Oil Base for Topical Drug Delivery

**DOI:** 10.3390/gels9020096

**Published:** 2023-01-22

**Authors:** Aidawati Mohamed Shabery, Riyanto Teguh Widodo, Zamri Chik

**Affiliations:** 1 Department of Pharmacology, Faculty of Medicine, Universiti Malaya, Kuala Lumpur 50603, Malaysia; 2 Department of Pharmaceutical Technology, Faculty of Pharmacy, Universiti Malaya, Kuala Lumpur 50603, Malaysia; 3 Universiti Malaya Bioequivalence Testing Centre (UBAT), Faculty of Medicine, Universiti Malaya, Kuala Lumpur 50603, Malaysia

**Keywords:** niosome, lidocaine, prilocaine, topical drug delivery, local anesthetic, nanoparticles

## Abstract

This study aimed to formulate semisolid niosomal encapsulated lidocaine and prilocaine using the patented palm oil base Hamin-C^®^ for further characterization and in vivo pain assessment. Seven formulations were initially studied with various chemical compositions. A thin-layer film hydration method was used to produce niosome using a mixture of surfactant (Span^®^ 40 or Span^®^ 60) and cholesterol (CHOL) at a 1:1 ratio, with/without a charge-inducing agent (diacetyl phosphate) (DCP) and with/without labrasol^®^. Niosome F1 formulation had been identified as the highest %EE achieved, at 53.74 and 55.63% for prilocaine and lidocaine, respectively. Furthermore, NIO-HAMIN F1 emulgel indicated the best formulation with higher permeability of prilocaine and lidocaine compared to the rest of the formulations. The reformulation of optimization of NIO-HAMIN F1 emulgel using a cold process to NIO-HAMIN F1-C emulgel to improve the viscosity resulted in higher diffusion of prilocaine and lidocaine by 5.71 and 33.38%, respectively. In vivo pain perception studies by verbal rating score (VRS) and visual analogue score (VAS) on healthy subjects show a comparable local anesthetic effect between NIO-HAMIN F1-C emulgel and EMLA^®^ cream.

## 1. Introduction

Local anesthetics are used clinically to reduce or eliminate pain whether for surgery or the management of other acute and chronic pain conditions [1]. Local anesthesia can be in the form of an injection or topical application. However, injectable anesthesia can be painful and hard to use, especially for children and patients with needle phobia [2]. Therefore, the topical application of local anesthetics is preferable in several medical procedures such as minor surgery because it is convenient, effective, and easy to apply [3].

**Figure 1:**
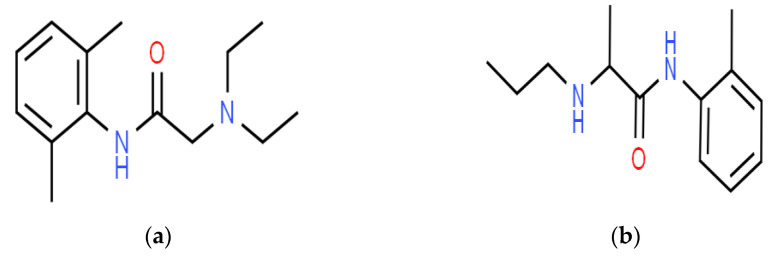
Chemical structures of (**a**) lidocaine and (**b**) prilocaine.

The prototypical amide-type local anesthetic lidocaine (Figure 1a), which was introduced by Lofgren in 1943, has a rapid onset of action, inherent potency, and moderate duration of action [4]. Many over-the-counter topical anesthetics contain lidocaine whether in a eutectic mixture or as monotherapy in the form of a gel, ointment, or patches [5]. Prilocaine (Figure 1b) is also an amide-type local anesthetic that is frequently used for regional pain relief through the injectable and transdermal route. Transdermal administration is preferable to injection due to it being self-administered and painless [6]. Prilocaine is mostly used in dentistry as an injectable form in combination with lidocaine as a topical preparation; it is used for dermal anesthesia and paresthesia. It shows low cardiac toxicity and is very commonly used in intravenous regional anesthesia. Prilocaine is slightly less potent than lidocaine but is considerably less toxic, produces less tissue vasodilation, and can be used reliably in plain solution form for short-duration procedures [7]. 

Astra Pharmaceuticals, USA, produced EMLA^®^, cream which is considered the gold standard for other topical anesthetics and has proven to have anesthetic efficacy in several clinical trials [2]. EMLA^®^ cream is formulated in an emulsion form, containing 2.5% lidocaine and 2.5% as a eutectic mixture in its oil phase [3]. Alireza et al. [8] have shown that the eutectic system of EMLA^®^ cream is efficient in providing an anesthetic effect for topical application.

Topical anesthetics required 30–60 minutes of dwell time after application before anesthetic effects took place [9]. Including EMLA^®^ cream, dwell times of 60 min are not very efficient, especially in emergency cases, and this will limit its clinical usage. Therefore, new topical anesthetics are commercially available to improve the onset of action, including ELA-Max, Amethocaine, and Topicaine. ELA-Max provides effective analgesia after 30 min of application, but in general, recommended application time for ELA-Max is 60 min, while Amethocaine has been shown to provide effective analgesia within 30–45 min and Topicaine’s recommended application time from the manufacturer is 30–60 min before venipuncture [10]. Three topical anesthetics can be alternatives to EMLA^®^ cream in terms of effectiveness, namely, tetracaine, liposome-encapsulated tetracaine, and liposome-encapsulated lidocaine [2].

The effectiveness of a topical local anesthetic can be affected by its delivery system. A topical delivery system of local anesthetics may be different in formulations and presentations, whether solid (patches), semisolid, or liquid [1]. Good local anesthetic topical delivery systems are characterized by less concentration of the drugs, better permeability and absorption, keeping the drugs at the target site longer, decreasing the clearance, and limiting local and systemic toxicity [11]. A novel means of improving drug delivery is with vesicular systems. A vesicular system is able to enhance the bioavailability of encapsulated drugs and provides therapeutic activity in a controlled manner for a prolonged period [12].

Vesicular systems consist of vesicular carriers such as soft lipid vesicles. Niosomes are one type of soft lipid vesicles that are elastic and deformable, which are used to encapsulate local anesthetics for dermal delivery efficiency and hence are able to slowly release large doses of local anesthetic agents to provide a prolonged period of analgesia without toxicity [1]. Moreover, niosomes can solve the issues related to insolubility, instability, low bioavailability, and rapid degradation of drugs [13]. They also can be easily and reliably produced in the laboratory compared to liposomes [14]. Niosomes are more stable and may provide faster penetration to the stratum corneum than liposomes [10].

Hamin-C^®^ palm oil base is a self-emulsifying base; it is a mixture of hydrogenated palm kernel oil and hydrogenated palm kernel stearin [15], making it more thermally stable and robust to the temperature changes. Therefore, Hamin-C^®^ palm oil base becomes a new revolution in the drug delivery system for semisolid and suppositories. In previous study, Hamin-C^®^ palm oil base has proved to be successful in delivering aspirin systemically via suppository formulation, in the testosterone transdermal delivery system (TDDS) in an animal model, producing the hypoglycemic effect for glucose control in rabbits and delivering lidocaine, which is comparable to EMLA^®^ cream [2]. Hamin-C^®^ palm oil base, which contains 5% lidocaine, demonstrates the cumulative amount of lidocaine release and is slightly higher than EMLA^®^ cream. Clinical studies also showed that Hamin-C^®^ palm oil base produces adequate numbness and is comparable to EMLA^®^ cream [2].

This study aimed to prepare niosome-encapsulated prilocaine and lidocaine loaded in Hamin-C^®^ palm oil base for topical anesthetic application. To increase the diffusion of lidocaine and prilocaine through the membrane, a smaller size vesicle in the form of a niosome was produced using the thin-film hydration method with some modifications. 

## 2. Results and Discussion

### 2.1. Niosome Encapsulation Efficiency (%EE)

All formulated niosome-encapsulated drugs were subjected to %EE to evaluate the amount of drug encapsulated in the niosome. Table 1 shows the %EE for all niosome. The highest %EE was achieved for formulations F1 and F6. Formulation F1, which was formulated using Span^®^ 40, showed greater encapsulation efficiency, with 53.74% and 55.63% for lidocaine and prilocaine, respectively. Formulation F6, which contains Span^®^ 60, gives a slightly lower %EE because its hydrophobic alkyl chain is shorter than in Span^®^ 40 [16].

Additional negatively charged DCP in formulations F1 and F6 resulted in higher %EE and lower %EE in F3, F4, and F5. This result showed that additional DCP alters encapsulation efficiency but also depends on other factors such as surfactant alkyl-side chain [17].

### 2.2. Niosome Size, Zeta Potential, and Polydispersity Index (PDI)

Size of vesicles or nanoparticles is very crucial in the transdermal drug delivery system. The size must be below 300 nm in order to be able to pass through the deepest skin layer. PDI values that are close to zero show less degree of heterogeneity of the particle size [18]. Zeta potential is important in determining the stability of the niosome, and higher values indicate a more stable niosome [19,20]. 

The lowest niosome size was achieved in F2, at 362.2 nm, and the highest was 4091 nm for F6. Niosome F2 formulation used Span^®^ 40 as a surfactant, while niosome F6 used Span^®^ 60. F2 does not contain DCP, while F6 contains 0.01 M DCP (Table 1). Span^®^ 60 produces a larger size niosome due to the longer alkyl group that produces a wider bilayer, resulting in a larger vesicle [21].

Niosome formulated using Span^®^ 40 seems to have a lower particle size if compared to Span^®^ 60 without labrasol, except for F7. Niosome F7 was sonicated at higher amplitude, thus decreasing its size. Niosome formulated using Span^®^ 60 was not added with labrasol because it will increase the vesicle size [22]. Since the method used to prepare niosome is film hydration, multilamellar vesicles (MLV) should be produced with a size in the range of 0.5–10 µm [23]. However, the size of the niosome produced in this study was in the range of 0.3–4.0 µm due to the use of probe sonification [24].

Niosome formulations F2, F5, and F6 were within negative zeta potential values ranging between −41.7 and −58.4 mv, which are sufficiently high for the electrostatic stabilization of niosomes [23]. F1 has a lower zeta potential, which was −28.4 mv, and is considered stable but with incipient instability. While other formulations have very high zeta potential, this shows very good stability [25]. The polydispersity index (PDI) of all formulations was more than 0.3 and did not correspond to a homogeneous population of colloidal systems [23]. All niosome formulation was characterized by a polydispersed population of particles, with the lowest value of PDI shown by F6 at 0.533, while F3, F4, and F5 have the highest PDI value, between 0.9–1.0, which is considered highly polydisperse with multiple particle size populations [26,27,28].

### 2.3. In Vitro Permeability Test

**Figure 2:**
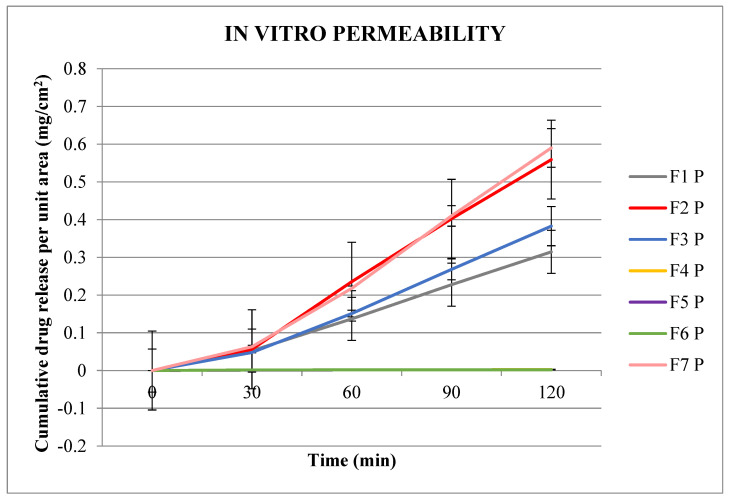
In vitro permeability of prilocaine for the various formulation of NIO−HAMIN emulgels. (P: prilocaine; L: lidocaine).

**Figure 3:**
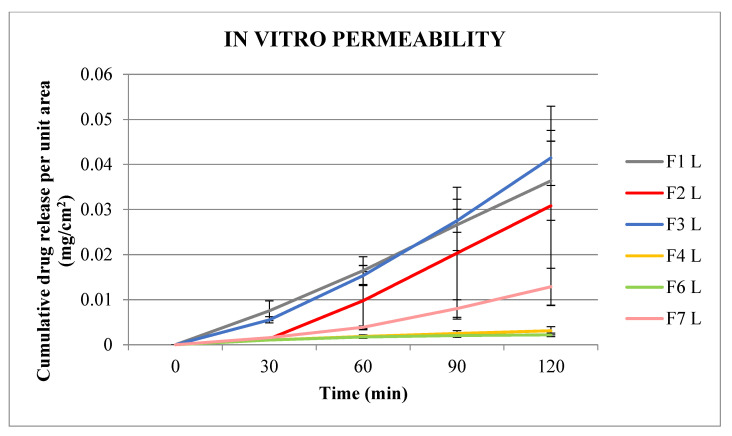
In vitro permeability of lidocaine for the various formulation of NIO-HAMIN emulgels. (P: prilocaine; L: lidocaine).

The cumulative prilocaine and lidocaine drug permeability per unit area from NIO-HAMIN emulgels across Strat M^TM^ membrane over 120 min were plotted on two separate scales, as shown in Figure 2 and Figure 3.

Figure 2 plots show that after 30 min, the cumulative amount of prilocaine that permeated the membrane was higher for NIO-HAMIN F1, F2, F3, and F7 as compared to NIO-HAMIN F4, F5, and F6. NIO-HAMIN F2 and F7 show consistently higher amounts of prilocaine compared to NIO-HAMIN F1 and F3 over a 2 h period (Figure 2). With a vesicle size of less than one micron in NIO-HAMIN F1, F2, and F7, a higher amount of prilocaine was diffused through the membrane. Additional labrasol^®^ in NIO-HAMIN F3 does enhance the permeability of prilocaine, although the size is larger than other formulations. This was attributed to the fact that labrasol^®^ is a nonionic surfactant capable of improving the solubility of prilocaine, while NIO-HAMIN F4, F5, and F6 give lower prilocaine permeability because the amount of niosome added in Hamin-C^®^ was only 2.5%. Additionally, the size of the vesicle is also larger if compared to other formulations [29].

Figure 3 shows that the cumulative amount of lidocaine at 30 min achieved for NIO-HAMIN F1 and F3 was higher compared to NIO-HAMIN F2, F4, F6, and F7. However, NIO-HAMIN F2 shows higher permeability at 60, 90, and 120 min compared to NIO-HAMIN F4, F6, and F7 but is still lower compared to NIO-HAMIN F1 and F3. 

All formulas have a lower amount of lidocaine permeability as compared to prilocaine, although the amount added into the donor compartment of the Franz diffusion cell was the same as prilocaine. This is probably due to the ability of lidocaine to permeate through the membrane since it is lower than the prilocaine, or because the release of lidocaine from the niosome is lesser compared to the prilocaine. In other words, lidocaine may have a stronger affinity with the niosome vesicles. This will be further investigated for the continuous development of NIO-HAMIN.

Since NIO-HAMIN F1 has the highest %EE and high prilocaine and lidocaine permeability compared to the other formulations, NIO-HAMIN F1 was selected for this study for further optimization. A high %EE leads to a high drug load in the emulgel; therefore, less emulgel needs to be applied onto the skin to obtain a high anesthetic effect. 

**Figure 4:**
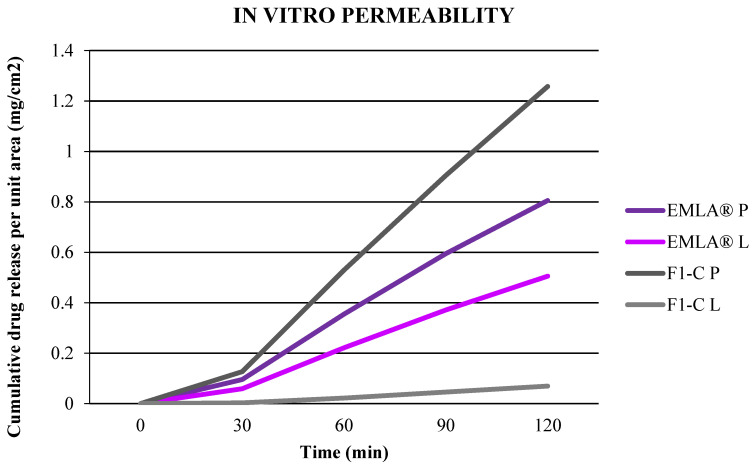
In vitro permeability of prilocaine and lidocaine of NIO-HAMIN F1-C emulgel after reformulated using a cold process method VS EMLA^®^ cream. (P: prilocaine; L: lidocaine).

To improve NIO-HAMIN F1 permeability, it was reformulated and optimized to become NIO-HAMIN F1-C using a cold process to adjust the pH of the emulgel to 9 since local anesthetics give a more rapid onset of action and are longer-acting in the nonionized-form at more basic pH [30]. This is because NIO-HAMIN emulgel from the hot process method will become watery after adjusting to pH 9 due to the decreasing viscosity of carbopol polymer in the presence of excess electrolytes [31]. As the results demonstrated, prilocaine in vitro permeability of NIO-HAMIN F1-C shows 5.71% higher permeability, while lidocaine permeability was 33.38% higher after 30 min (Figure 4) when comparing to NIO-HAMIN F1 (hot process). With a high permeability rate in NIO-HAMIN F1-C, this may lead to a high effective rate to gain an anesthetic effect at the site of application.

### 2.4. NIO-HAMIN F1-C Emulgel Characterization

Physical characterizations of NIO-HAMIN F1-C emulgel are summarized in Table 2. The mean pH of the emulgel is 8.86 ± 0.44, which is alkaline. The emulgel color was measured using CIE LAB color space. The L* value indicates the lightness, the a* value indicates the color between red to green, and the b* value the color between yellow to blue [32]. Using CIE LAB color calculator (http://colorizer.org/, accessed on 27 November 2020), one can conclude that the cream is slightly whitish grey.

**Figure 5:**
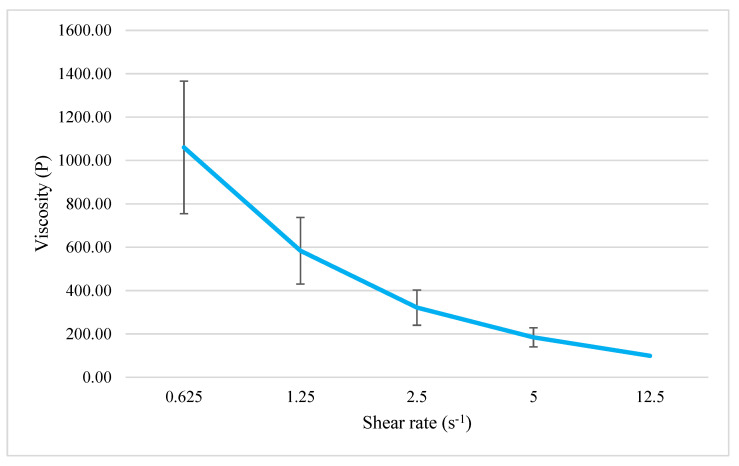
Influence of shear rate on viscosity of NIO-HAMIN F1−C emulgel. Data presented as mean ± SD (*n* = 3).

**Figure 6:**
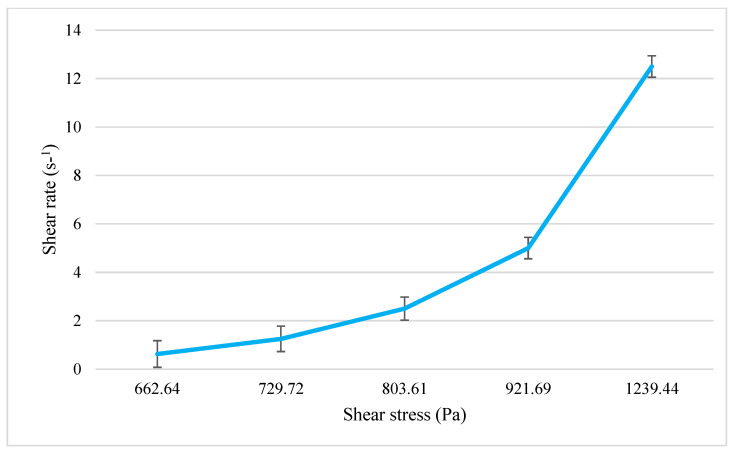
Rheogram of NIO-HAMIN F1−C emulgel: shear stress as a function of shear rate. Data presented as mean ± SD (*n* = 3).

The viscosity and rheogram of the NIO-HAMIN F1-C emulgel are shown in Figure 5 and Figure 6. The shear stress of the emulgel increased as the shear rate increased. The viscosity of the emulgel decreased as the shear rate increased, which shows that the emulgel is a pseudoplastic exhibiting non-Newtonian flow behavior [33]. This shear-thinning behavior makes the emulgel easy to apply and stays static when rubbed on the skin. 

Drug content for NIO-HAMIN F1-C emulgel was analyzed using a developed HPLC to study the consistency of the content, and the results were consistent at 25.21 ± 0.53 mg/g emulgel (*n* = 10). 

### 2.5. In Vivo Pain Assessment

Verbal rating score (VRS) and visual analogue score (VAS) [34,35] were adopted as the methods to assess the efficacy of NIO-HAMIN F1-C emulgel as a topical local anesthetic preparation in comparison against EMLA^®^ cream, with the results as discussed below. 

#### Study Design and Subject Admission

The clinical study was conducted in two phases:

(1) Study 1

**Figure 7:**
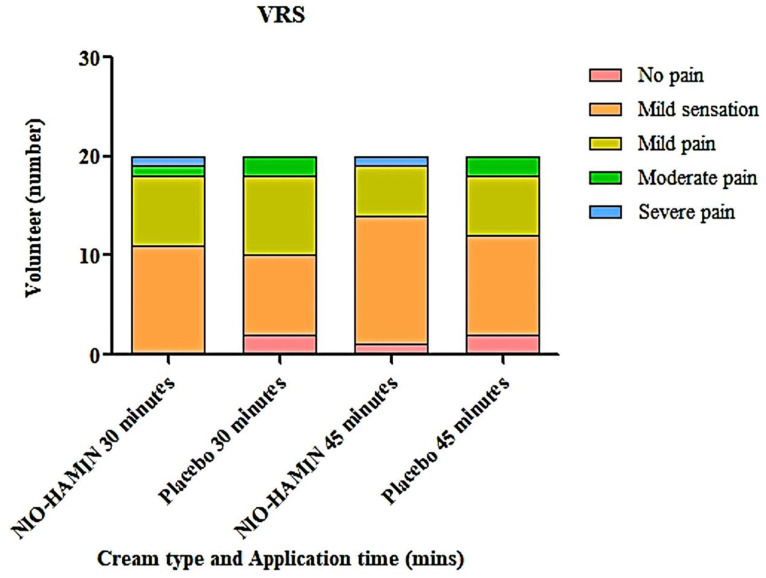
Verbal rating score (VRS) for NIO-HAMIN F1-C emulgel compared to placebo. Values are presented as number of subjects vs. treatment (*n* = 20, *p* < 0.05).

**Figure 8:**
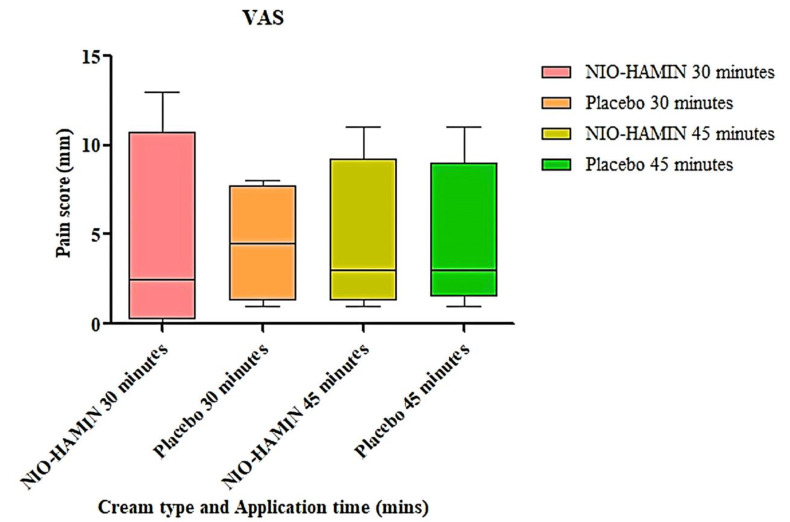
Visual analogue score (VAS) for NIO-HAMIN F1-C emulgel compared to placebo. Values are presented as pain score vs. treatment (*n* = 20, *p* < 0.05).

Figure 7 and Figure 8 show the VRS and VAS scores for the 20 subjects who participated in the study. Statistical analysis showed that there was no significant difference between NIO-HAMIN and placebo for both application times applied (*p* < 0.05).

**Figure 9:**
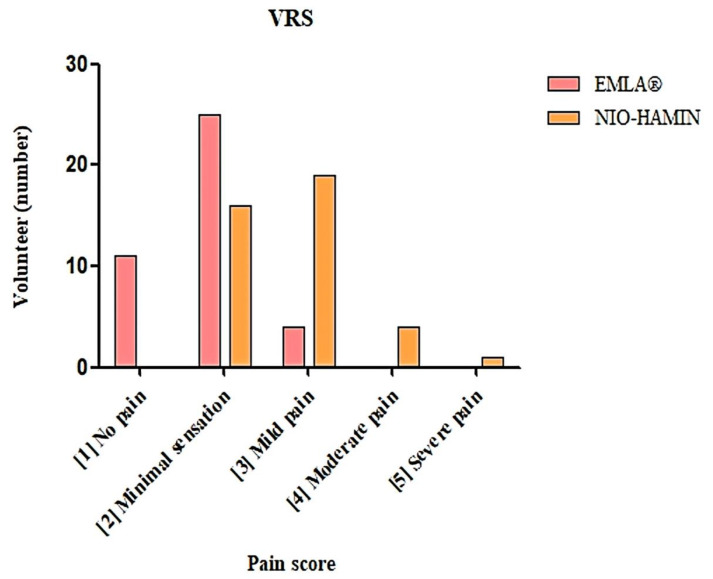
Verbal rating score (VRS) for NIO-HAMIN F1-C emulgel compare to EMLA^®^ cream. Values are presented as the number of subjects vs. pain score (*n* = 40, *p* < 0.05).

However, the distribution of pain scores was reduced by an increase in the application time of NIO-HAMIN such as from 30 min to 45 min. On the contrary, placebo pain score distribution increased with the increased application time (Figure 9). Since there was no significant difference between the pain score of NIO-HAMIN and placebo, for study 2, application time was decided at 60 min, which is the recommended application time for EMLA^®^ cream [36].

(2) Study 2

**Figure 10:**
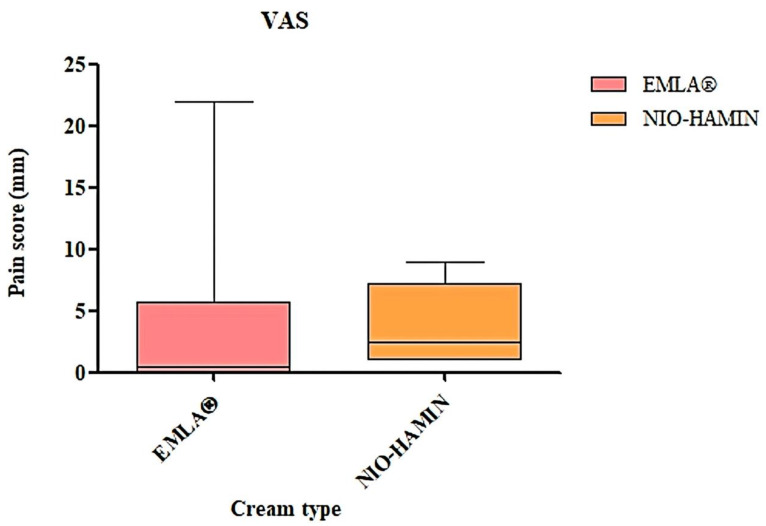
Visual analogue score (VAS) for NIO-HAMIN F1-C emulgel compared to EMLA^®^ cream. Values are presented as pain score vs. treatment (*n* = 40, *p* < 0.05).

In total, 40 healthy subjects were involved in the study. The VRS result showed that 11 subjects scored no pain for EMLA^®^ cream, while none did so for NIO-HAMIN F1-C emulgel. A total of 25 subjects scored minimal sensation for EMLA^®^ cream and 16 subjects did so for NIO-HAMIN F1-C emulgel. Most of the subjects scored mild pain for NIO-HAMIN F1-C emulgel (*n* = 19). There were four subjects who scored moderate pain and one subject who scored severe pain for NIO-HAMIN F1-C emulgel but none for EMLA^®^ cream. In reference to VRS, the statistical analysis of both pain scores (minimal sensation and mild pain) regarding NIO-HAMIN F1-C emulgel and EMLA^®^ were found significantly effective (*p* < 0.05). However, the result showed a significant difference for “No Pain” since none of the subjects scored no pain for NIO-HAMIN F1-C emulgel (*p* < 0.05) (Figure 10). VAS analysis for study 2 showed no significant difference or was significantly effective between EMLA^®^ cream and NIO-HAMIN F1-C emulgel with median differences of 4.35% (*p* < 0.05, *p* = ns) (Figure 10). 

## 3. Conclusions

In conclusion, we successfully formulated and characterized a new semisolid niosomal local anesthetic emulgel using a patented palm oil base, Hamin-C^®^, which contains lidocaine and prilocaine. This new formulation was tested and characterized by having good properties of topical local anesthetic and enhanced release of local anesthetic agents, in vitro. In vivo study shows that the NIO-HAMIN F1-C emulgel local anesthetic effect is comparable but slightly less effective as compared to EMLA^®^ cream. This is probably because the lidocaine release from NIO-HAMIN F1-C is less as compared to EMLA^®^ cream. However, the prilocaine released was higher for NIO-HAMIN F1-C compared to EMLA^®^ cream. More investigation and modification in the formulation are required to improve the release of lidocaine from the niosome. In this case, to improve the release we should have a smaller size with a lower polydispersity index of the niosome. These changes would probably produce a better in vivo effect. 

## 4. Materials and Methods

### 4.1. Materials

Lidocaine hydrochloride, Span^®^ 60, SPAN^®^ 40, and Diacetyl phosphate (DCP) were purchased from Sigma-Aldrich Co (St. Louis, MO, USA). Prilocaine hydrochloride was purchased from UTRC (Toronto, ON, Canada). Disodium hydrogen phosphate was purchased from R&M Chemicals (Essex, UK). Sodium dihydrogen phosphate anhydrous was purchased from QReC (Selangor, Malaysia). Sodium hydroxide was purchased from Sharlau Chemie (S.A, Barcelona, Spain). Cholesterol, 95% stabilized, was purchased from Acros Organics (Fair Lawn, NJ, USA). Acetonitrile and chloroform were purchased from Fisher Scientific (Fair Lawn, NJ, USA). Labrasol was purchased from Gattefosse (Cedex, France). Propylene glycol was purchased from Dow Chemical (Thailand). Hamin-C^®^ palm oil base was purchased from Oleopharma Sdn. Bhd., (Malaysia). Acrylates/C10–30 Alkyl Acrylate Crosspolymer was purchased from Lubrizol (Clifton, NJ, USA). Strat M™ membrane was purchased from Merck (Darmstadt, Germany).

### 4.2. Methods

#### 4.2.1. Preparation and Evaluation of Niosome-Encapsulated Prilocaine and Lidocaine

Niosome was prepared using a thin-film hydration technique. Surfactant (Span^®^ 40 or 60) and cholesterol were composed in a molar ratio of 1:1. The composition of surfactant, cholesterol, labrasol, Diacetyl phosphate, lidocaine, and prilocaine was dissolved in chloroform according to the formulation code as summarized in Table 3. Thin-layer film was achieved by rotary evaporation (EYELA, Rikakika, Tokyo, Japan) of the mixture at 60 °C. Phosphate buffer (PBS) pH 6 was added to the thin-layer film to hydrate it. The niosome was ultrasonicated at 40/60 amplitudes with 2000 J energy and 30 W powers for 60 min or 120 min using an ultrasonic homogenizer (Biologics, Inc., Bristow, VA, USA) with a titanium probe to agitate particles. Niosome then was centrifuged (Sartorius, Goettingen, Germany) at 11,000 rpm for 60 min to remove the excess drug. The supernatant was then removed, and distilled water was added and vortex mixed (Snijders Scientific, Tilburg, Holland) and ultrasonicated (Kudos, Shanghai, China) to resuspend the niosome. The finished niosome was stored at a refrigerator temperature between 4 °C to 8 °C before further use. Table 1 summarizes the composition of niosomes for various formulations prepared.

#### 4.2.2. Encapsulation Efficiency

Niosome was mixed with Isopropyl alcohol (IPA) at a 1:10 ratio and vortex mixed until a clear solution was obtained. The clear solution was filtered, and the quantification of drug content was analyzed using a developed high-performance liquid chromatography (HPLC) as described below. Encapsulation efficiency (EE) percentage was calculated using the following formula:

%EE = (Total drug content/Total drug added) × 100 × df

where

1. Total drug content = amount of drug obtained from HPLC analysis;
2. Total drug added = amount of drug added into the formulation;
3. df = dilution factor.

#### 4.2.3. Development of High-Performance Liquid Chromatography (HLPC) for Lidocaine and Prilocaine Analysis

HPLC (Shimadzu, Kyoto, Japan) series LC 20AD was used in this study. Phenomenex Synergi^TM^ 4 μm Fusion-RP 80 Å, with dimensions of 4 μm, 150 × 4.6 mm C18 column, was used as the stationary phase, while 0.01 M phosphate buffer pH 6 and acetonitrile at 55:45 ratio was used as the mobile phase. The injection volume was set to 10 μL, and the analysis was run at a flow rate of 1 mL/min for 8 min. The temperature of the HPLC system was maintained at 25 °C. Lidocaine and prilocaine were detected at 210 nm using a UV detector [4]. The standard curve showed good linearity with regression of coefficient (r^2^) greater than 0.998. The retention times were 3.10 and 4.10 min for prilocaine and lidocaine, respectively. The limit of detection (LOD) for prilocaine and lidocaine were 0.3 mg/L and 0.4 mg/L, respectively, while the limit of quantification (LOQ) was 1.0 mg/L and 1.2 mg/L, respectively.

#### 4.2.4. Niosome Particle Size, Zeta Potential, and Polydispersity Index (PDI)

The size and zeta potential of niosome-encapsulated lidocaine and prilocaine were measured using a Malvern Zetasizer (Malvern Panalytical, Malvern, UK). A total of 100 µL of niosomes were pipetted and diluted in 900 µL of distilled water. All measurements were taken at 25 °C.

#### 4.2.5. Preparation of Niosome Encapsulated Lidocaine and Prilocaine NIO-HAMIN Emulgel

##### Preparation of NIO-HAMIN Emulgel by Hot Process

Hamin-C^®^-based cream was prepared according to Khamdiah Khodari et al. [4]. The water phase consists of distilled water, propylene glycol (PG), and sodium hydroxide, 10% (NaOH), while the oil phase containing Hamin-C^®^ base oil and carbomer was heated separately to 40–50 °C. Once both phases’ temperatures were achieved, the oil phase was added to the water phase while stirring using an overhead stirrer (IKA, Germany) at gradual speed from 400 to 800 rpm. Once the emulgel formed, the speed was stopped gradually until it cooled down. Lastly, the base emulgel was transferred into a container and left at room temperature for 24 h to stabilize the whole texture. All the ingredients involved were weighed in % *w*/*w* [4]. 

Hamin-C^®^-based emulgel from the above preparation was weighed and stirred while mixed with niosome at different percentages (97.5% for F1 and F7, 95% for F4–F6 and 50% for F2 and F3) to form NIO-HAMIN emulgels. Different percentages of niosome were used to find the right percentage of drug content to achieve the highest diffusion amount using the Franz diffusion test. 

##### Preparation of NIO-HAMIN Emulgel by Cold Process

Niosome, propylene glycol, and carbomer were weighed separately and mixed by stirring slowly until the carbomer was wetted. Sodium hydroxide (10%) was added to form a gel. Hamin-C^®^ palm oil base was added to the gel and the mixture was stirred until all the Hamin-C^®^ bases were thoroughly mixed. The preservative was added and stir-mixed before the pH of the emulgel was adjusted to 9 with 10% NaOH.

#### 4.2.6. In Vitro Skin Permeation Test

A Franz diffusion cell (Hanson, California, USA) was used to evaluate in vitro skin permeation of the NIO-HAMIN local anesthetic emulgel preparations, with EMLA^®^ cream used as a reference. Strat M™ membrane (Merck, Darmstadt, Germany) was selected in this study because it correlates more closely to human skin than any other synthetic membrane. The surface area of the membrane was fixed at 1.767 cm^2^ and phosphate buffer at pH 6 as the medium. About 1 g of sample was loaded into the donor compartment and left for 2 h. The period of sample collection was every 30 min. The system was performed under a controlled temperature of 37 °C. The collected samples were analyzed using a developed HPLC method as described in 2.2.3. The permeability profile was demonstrated using the following calculation [2]:

Q = Cn·V/A

where

1. Q = cumulative amount of drug release;
2. Cn = concentration of lidocaine/prilocaine in the receiver compartment (μg/mL);
3. V = volume of the receiver compartment;
4. A = surface area of the membrane in cm^2^.

#### 4.2.7. Physicochemical Characterizations

The physicochemical characterization of an optimized NIO HAMIN emulgel covered the determination of color, pH, rheology, and viscosity properties. The color was determined using a color spectrophotometer (Minolta, Japan) with parameters a* = green-to-red ratio, b* = yellow-to-blue ratio, and L* = light-to-dark ratio measured. The pH was measured using a pH meter (Eutech Instrument, Singapore). Rheology/viscosity properties were evaluated at room temperature using a viscometer (Brookfield, Stoughton, WI, USA) with spindle no. S29 at 2.5–50 RPM for 30 s.

#### 4.2.8. Drug Uniformity Content

A total of 1 g of an optimized NIO-HAMIN emulgel was weighed and extracted using 15 mL methanol with the aid of ultrasonication for 15 min. The solutions were filtered into a 25 mL volumetric flask; Isopropyl alcohol was used to top up to the mark [37]. The prepared sample was analyzed using a developed HPLC, as described in 4.2.3.

#### 4.2.9. In Vivo Pain Assessment

The efficacy of an optimized NIO-HAMIN emulgel as a topical local anesthetic through human skin and to produce numbness at the applied area was assessed in 60 healthy adult subjects. Two methods of pain assessment were adopted, namely, the verbal rating score (VRS) and the visual analogue score (VAS) [34]. VRS assessment was conducted according to the severity of the pain. Subjects were provided with a choice of five answers: no pain, minimal sensation, mild pain, moderate pain, and severe pain [35]. On the other hand, VAS was performed by designing a 100 mm horizontal line and marks of “no pain” at the end line and “severe pain” at the other end of the line were given. Subjects were requested to make a vertical cross on the line which relates to the intensity of pain experienced during the procedure.

#### 4.2.10. Study Design and Subject Admission

##### The First Phase of the Clinical Study (Study 1)

Study 1 was conducted to determine the onset of action of an optimized NIO-HAMIN emulgel in 20 healthy adult subjects with normal skin conditions. The study was designed as one that is single-blinded and placebo-controlled. The ventral aspect of the forearm of the right and left upper limbs was marked with an area of 10 cm^2^ at two sites. Approximately 2 g of an optimized NIO-HAMIN emulgel and 1 g of EMLA^®^ cream were applied for 30 and 45 min. Upon 5 min removal of the two applied preparations, 20 pinpricks were performed dispersedly on the mark site [4]. Subjects rated the pain they experienced using VAS and VRS pain assessment as described above.

##### The Second Phase of the Clinical Study (Study 2)

Study 2 was conducted to evaluate the effectiveness of an optimized NIO-HAMIN emulgel as a local anesthetic preparation in comparison against EMLA^®^ cream, after the application period as decided in the study 1, using the same pain assessment methods as above. The study design was a single-dose, blinded, crossover, randomized, and balanced study on 40 healthy adult subjects with normal skin condition. The study was carried out after receiving approval from the Universiti Malaya Medical Ethics Committee (MREC ID No.: 202032-8335).

#### 4.2.11. Statistical Analysis

A Student’s *t*-test and Wilcoxon signed ranked test were used to analyze the differences in means and clinical study data, respectively. Test results with *p* < 0.05 were considered to be significant.

## Figures and Tables

**Table 1:** Niosome encapsulated lidocaine and prilocaine percentage of entrapment efficiency (%EE), particle size (nm), zeta potential (mv), and polydispersity index (PDI).

Formulation Code	Vesicular Size (nm)	PDI	Zeta-Potential	%EE	
				LDC	PLC
F1	748.6	0.732	−28.4 ± 18.2	53.74 ± 0.03	55.63 ± 0.03
F2	362.2	0.697	−54.2 ± 12.3	34.36 ± 0.03	32.63 ± 0.03
F3	1206	1.000	−66.3 ± 13.4	27.60 ± 0.04	29.51 ± 0.04
F4	1035	0.945	−62.8 ± 12.4	24.36 ± 0.03	26.83 ± 0.03
F5	1902	0.956	−50.3 ± 6.33	26.45 ± 0.08	28.23 ± 0.08
F6	4091	0.533	−46.8 ± 8.29	50.26 ± 0.09	47.73 ± 0.08
F7	623.7	0.885	−72.9 ± 24.9	8.94 ± 0.03	12.27 ± 0.03

**Table 2:** NIO-HAMIN F1-C emulgel formulation characterization.

Parameter	NIO-HAMIN F1-C Emulgel			Mean	±SD
	Day 0	Month 1	Month 2		
pH	9.36	8.53	8.67	8.86	0.44
Viscosity (P)					
2.5 rpm	406.67	1244.67	1529.33	1060.22	583.62
5 rpm	242.67	675.33	833.33	583.78	305.79
10 rpm	147.67	376.67	440.00	321.44	153.79
20 rpm	91.83	217.00	244.18	184.34	81.26
50 rpm	51.87	106.80	138.80	99.16	43.97
Color					
L*	85.52	86.00	85.49	85.67	0.29
a*	−0.70	−0.95	−1.24	−0.96	0.27
b*	5.02	5.54	4.62	5.06	0.46

**Table 3:** Composition of the niosome in various formulations.

Formulation Code	Lidocaine (g)	Prilocaine (g)	SURFACTANT (M)		Cholesterol (M)	Labrasol (%)	DCP (M)
			Span^®^ 40	Span^®^ 60			
F1	0.25	0.25	1	-	1	2	0.01
F2	0.25	0.25	1	-	1	2	-
F3	0.50	0.50	1	-	1	2	0.01
F4	0.25	0.25	1	-	1	-	0.01
F5	0.25	0.25	-	1	1	-	0.01
F6	0.25	0.25	-	1	1	-	0.01
F7	0.25	0.25	-	1	1	-	-

## Data Availability

data is unavailable due to privacy or ethical restrictions.
